# Functional electrical stimulation mediated by iterative learning control and 3D robotics reduces motor impairment in chronic stroke

**DOI:** 10.1186/1743-0003-9-32

**Published:** 2012-06-07

**Authors:** Katie L Meadmore, Ann-Marie Hughes, Chris T Freeman, Zhonglun Cai, Daisy Tong, Jane H Burridge, Eric Rogers

**Affiliations:** 1 School of Electronics and Computer Science, University of Southampton, Southampton, SO17 1BJ, UK; 2 Faculty of Health Sciences, University of Southampton, Southampton, SO17 1BJ, UK

**Keywords:** Functional electrical stimulation, Upper limb, Stroke rehabilitation, Iterative learning control, Robotic support, Virtual reality

## Abstract

**Background:**

Novel stroke rehabilitation techniques that employ electrical stimulation (ES) and robotic technologies are effective in reducing upper limb impairments. ES is most effective when it is applied to support the patients’ voluntary effort; however, current systems fail to fully exploit this connection. This study builds on previous work using advanced ES controllers, and aims to investigate the feasibility of Stimulation Assistance through Iterative Learning (SAIL), a novel upper limb stroke rehabilitation system which utilises robotic support, ES, and voluntary effort.

**Methods:**

Five hemiparetic, chronic stroke participants with impaired upper limb function attended 18, 1 hour intervention sessions. Participants completed virtual reality tracking tasks whereby they moved their impaired arm to follow a slowly moving sphere along a specified trajectory. To do this, the participants’ arm was supported by a robot. ES, mediated by advanced iterative learning control (ILC) algorithms, was applied to the triceps and anterior deltoid muscles. Each movement was repeated 6 times and ILC adjusted the amount of stimulation applied on each trial to improve accuracy and maximise voluntary effort. Participants completed clinical assessments (Fugl-Meyer, Action Research Arm Test) at baseline and post-intervention, as well as unassisted tracking tasks at the beginning and end of each intervention session. Data were analysed using *t-*tests and linear regression.

**Results:**

From baseline to post-intervention, Fugl-Meyer scores improved, assisted and unassisted tracking performance improved, and the amount of ES required to assist tracking reduced.

**Conclusions:**

The concept of minimising support from ES using ILC algorithms was demonstrated. The positive results are promising with respect to reducing upper limb impairments following stroke, however, a larger study is required to confirm this.

## **Background**

Stroke is a leading cause of death and disability in the UK, and about 50% of people who survive a stroke require some form of rehabilitation to reduce impairment and assist with activities of daily living [1-3]. Upper limb function is particularly important in regaining independence following stroke; impairments impact on daily living and well-being [4,5].

Research has consistently identified treatment intensity and goal oriented strategies as critical elements for successful therapeutic outcomes [6-10]. To further maximise rehabilitation effects, novel therapeutic and cost-effective rehabilitation interventions need to be developed and may combine different methodological techniques. For example, the combined use of electrical stimulation (ES), robot-aided therapy and virtual reality (VR) environments has been suggested to be particularly promising with respect to upper limb rehabilitation in chronic stroke [10,11].

Following stroke, robot and ES therapies have been demonstrated to reduce upper limb motor impairments [6,7,10,12-14]. Furthermore, these techniques have been highlighted as a way to facilitate the intensity of the training received [10], and allow training despite muscle weakness and without the aid of a therapist. In addition, when used with a real-time system which displays the participants’ arm and hand movements in a VR environment, the practiced movements can be very task-specific [11,15]. These types of technologies may be more easily transferred into patients’ homes, increasing the intensity and task specificity of the training and reducing the time and expense constraints on therapists [16].

The therapeutic effect of ES in rehabilitation is known to increase when associated with a person’s voluntary effort [12]. However, a disadvantage of many ES approaches is that they fail to encourage voluntary contribution. In addition, the vast majority of upper limb stroke patient trials using ES employ open-loop or triggered controllers [12,17], which can lead to imprecise control of movement. In the few cases that closed-loop control has been employed, a simplistic structure and lack of a model means accurate performance is still rarely achieved [18]. Employed mainly with spinal cord injury patients, one of the few advanced control methodologies used comprises artificial neural networks [19,20]. However such model-free approaches have limited ability to adapt to changing physiological conditions, must be re-trained for use with different movements, and being of a “black-box” structure, do not permit stability and performance analysis.

The study reported in this paper investigates the feasibility and effectiveness of a novel 3D rehabilitation platform which combines robotic support, ES and VR. The system allows patients to receive the benefits of muscle-specific targeted ES within a tightly controlled, safe and motivating environment. In this platform, ES is mediated by iterative learning control (ILC), a technology transferred from industrial robotics which is applicable to systems which repeatedly perform a finite duration tracking operation [21]. After each repetition, ILC uses data gathered on previous executions of the task, often in combination with a model of the underlying system, to update the ES signal that will be applied on the subsequent trial. Hence ILC learns from previous experience the stimulation which maximises performance, and can effectively respond to changes in the model. ILC calculates the required control action in an optimal setting, allowing strict regulation of the amount of ES, its trial-to-trial variation, and the resulting movement error. Through use of appropriate weighting parameters a precise balance can be placed between encouraging voluntary effort and ensuring accurate movement [22,23].

ILC is one of very few model-based upper limb ES control methodologies that has previously been used in a clinical study [24-26]. During this study, stroke participants attended 18 intervention sessions of 1 hour duration in which they practiced planar reaching tasks, tracking a moving spot of light. These movements were assisted by ILC mediated ES applied to the triceps of the impaired arm. Unassisted tracking performance (i.e., movements without the aid of ES) improved over the course of the intervention and changes in muscle activation patterns towards those of unimpaired participants were also observed [24,25]. Whilst establishing the feasibility of advanced upper limb ES control approaches in the clinical domain, this planar system did not assist shoulder movement and by providing full mechanical support to the forearm, allowed very limited shoulder elevation.

**Figure 1:**
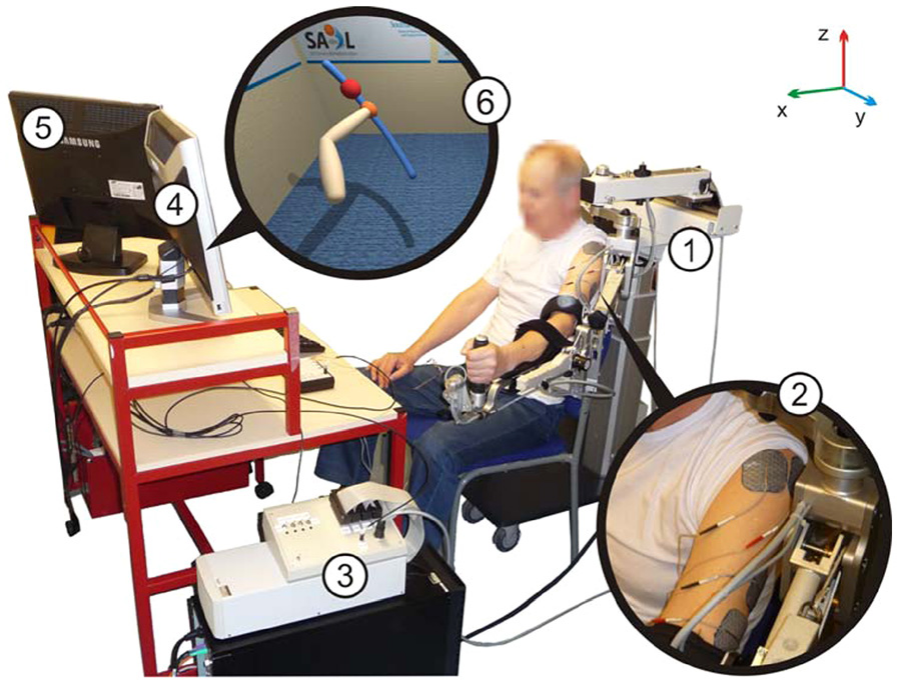
**SAIL system components:** 1) Hocoma ArmeoSpring® support, 2) surface electrodes on triceps brachii and anterior deltoid muscles, 3) realtime processor and interface module, 4) monitor displaying VR task, and 5) monitor displaying therapist user interface. 6) shows an example of a reaching task displayed to a stroke participant with left hemipshere damage. An image of their own arm is shown and they are encouraged to follow a sphere which moves along a reference path (the trajectory); in this case from bottom right to top left.

To address these limitations and increase the potential of this novel approach to stroke rehabilitation, a new system has been developed to assist participants in performing more functional, 3D reaching tasks with ES applied to triceps and anterior deltoid muscles [22,23]. Termed SAIL: Stimulation Assistance through Iterative Learning, this system comprises a commercial robotic arm support interfaced with custom-designed ES hardware and real-time ES control environment, together with a custom-made VR task display system (see Figure 1).

The commercial exoskeleton robot is a purely passive ‘un-weighing’ system which supports the patient’s arm against gravity via two springs incorporated into the mechanism. Each of its joints contains a resolver which records its angular position and this information is used by both the ES control system, and the VR task display. Whilst building on previous work, the ES controller incorporates substantial developments in terms of biomechanical modelling, identification, and control complexity compared with the planar system previously reported. In particular, a five degree-of-freedom biomechanical model of the combined human and robotic arm system was developed, along with identification procedures using kinetic, kinematic and ES input data which are suitable for patients [23,27]. Then parallel feedback and feedforward controllers were derived using techniques from nonlinear optimisation to achieve robust tracking whilst maintaining strict trial-to-trial bounds on the change in input, and the patients’ arm dynamics occurring along each trial [22,23,28,29]. Moreover, the muscle structures used in the model, identification procedure and controller have been specifically developed for application to stroke patients [27].

Preliminary tests to assess whether the ILC algorithms were accurately mediating the ES took place with unimpaired participants. Results confirmed that SAIL was effective in moving the arm to produce precise reaching movements, and that tracking performance improved over a series of trials see [22,28,29]. The aim of the study reported in this article was to assess the technological feasibility and rehabilitation effectiveness of the SAIL system with chronic stroke participants.

## Method

### Design

All participants attended 18 sessions at the University of Southampton, Faculty of Health Sciences. Data collection was carried out by an experienced researcher. Participants also attended three clinical assessment sessions that were carried out by independent assessors (a physiotherapist and psychologist); the Action Research Arm Test (ARAT; [30]) and Fugl-Meyer Assessment (F-M; [31]) and the cancellation subtests of the Behavioural Inattention Test ([32]; see [33]). All assessments were conducted according to standard protocol.

### Participants

A convenience sample of stroke participants was recruited from a volunteer list held by the Faculty of Health Sciences and from local stroke groups. Inclusion criteria were: i) aged 30–75 years; ii) ES produced movement without undue discomfort; iii) could comply with study protocol; iv) could communicate effectively; v) gave informed consent; vi) stroke causing hemiplegia for at least 6 months and vii) impaired upper limb that included an inability to effectively extend the elbow in reaching. Exclusion criteria were: i) any active device implant; ii) any metal implant in upper limb; iii) uncontrolled epilepsy; iv) pregnancy; v) any serious or unstable medical or psychological condition or cognitive impairment; vi) interpreter required; vii) participation in another upper limb physical rehabilitation study. Participants were recruited over two months from October to December 2010.

### Procedure

#### Preliminary session

Following University of Southampton, Faculty of Health Sciences ethical approval (FoHS ETHICS-2010-30) eight participants volunteered for the study. All participants gave written informed consent. A total of five participants were recruited to the study (the other three participants did not respond to the ES: inclusion criterion ii).

### Clinical outcome assessments

Prior to the intervention sessions, two assessments (set four weeks apart) were completed to establish baseline performance for three clinical outcome measures. Following the intervention sessions, a final assessment was conducted one or two days later.

Assessments of the upper limb consisted of the F-M and ARAT outcome measures, assessing impairment and function respectively. These are valid and reliable for use with stroke participants [30-32,34,35].

### Intervention sessions

**Figure 2:**
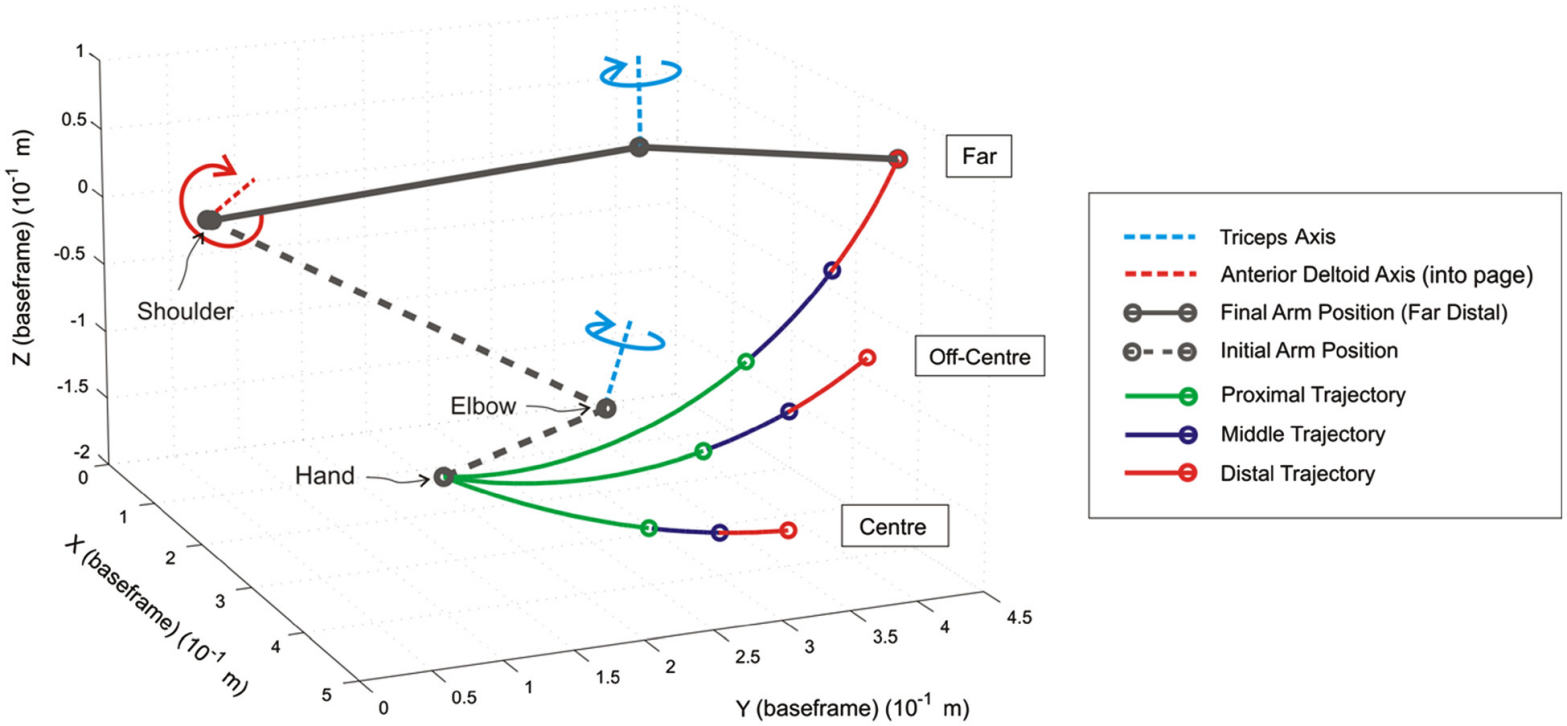
**Example of arm location, joint axes, and different trajectories.** Tracking task duration was 5 or 10 seconds.

During the intervention sessions, participants practiced reaching movements, moving their impaired arm to track a slowly moving ball along a specified trajectory displayed on a computer screen. As illustrated in Figure 2, there were 18 possible trajectories; each could be in one of three orientations relating to space in front and to the hemiplegic side (centre, off-centre and far), one of three lengths (proximal, middle, and distal) and one of two speeds (5 second and 10 second duration). To assist training of elbow extension and shoulder flexion and abduction, the impaired arm was supported by a robotic arm and ES was applied to the triceps and anterior deltoid. Between each trial, the ILC scheme modified the ES signal applied to each muscle, using data recorded over previous attempts together with the full dynamic model, in order to precisely assist tracking during the next attempt (see “Model Parameters” for a more detailed description of ILC). At the same time, the ILC scheme strictly controlled the level of ES assistance to encourage maximum voluntary contribution from the participant (see [22,23], for full details).

### Set up

#### Equipment and workspace

At the beginning of each session, the researcher identified each muscle and placed electrodes over the muscle body. The arm was then supported by the researcher, ES was applied to the muscle and the movement observed. The electrodes were adjusted as necessary until the best movement with the minimal ES was achieved. Participants were then seated at the workstation in front of two computer screens: one screen was viewed by the participant and the other by the researcher. The participant’s screen (which was located on the hemiplegic side) showed a VR environment displaying the trajectory to be tracked and a representation of the participant’s arm (that mirrored the participant’s movements in real-time; see Figure 1). This provided the participant with immediate visual feedback and facilitated motivation for the tracking task. The second screen displayed a custom graphical user interface which was used by the researcher to select the tasks and adjust the parameters used. The participant’s hemiplegic arm was loosely strapped into the support mechanism, which was adjusted so that the participant’s arm was fully supported off the knee (see Figure 1).

The frequency of stimulation was fixed at 40 Hz in all tests, with a pulsewidth controlled in real-time by the ILC algorithms. To identify ES amplitudes for both muscles, the pulsewidth was set at a maximum value and the participants gradually increased the ES amplitude applied to each muscle until they reached a comfortable level that produced movement. Note that although the participants controlled the ES amplitude, this was monitored by the researcher. The pulsewidth was then reduced to zero, and the stimulation amplitudes were then fixed for the remainder of the session to ensure participant comfort and safety. A workspace in which participants could extend to their full range of movement with assistance from ES was also established, by calculating the spatial coordinates from the highest point in ipsilateral space that the participant could reach when ES was applied to both muscle groups, the lowest point closest to the participant’s contralateral thigh, and a front point relating to elbow extension directly in front of the participant. In this way, the workspace related directly to the amount of movement produced by the ES.

### Model parameters

Parameters required for the dynamic model of the combined arm and support used by the ILC were also established (see [22] for full details). This involved firstly locating the axis about which the anterior deltoid produces movement, which was achieved by stimulating the muscle and then fitting a plane to the resulting movement of the elbow in 3D space using least squares optimisation [22] (Figure 2 shows an example of this axis, which is normal to the fitted plane). Using the measured lengths of the participant’s upper arm and forearm, the kinematic relationship between the arm’s position in Cartesian space and the vector of joint angles, Φ(t), could then be calculated, as well as the system Jacobian matrix, *J*(Φ)).

**Figure 3:**
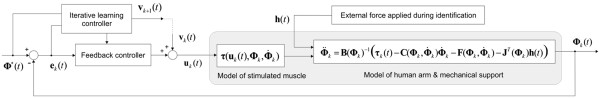
**Iterative learning control scheme used for ES showing model of the combined human arm and mechanical support used in the controllers.** The subscript ‘k’ denotes the iteration number, Φ(t) is the vector of human arm joint angles, $\Phi^{*}(t)$ is the vector of joint angles that the arm is required to follow, u(t) is the vector of stimulation pulsewidths applied to each muscle, and v(t) is the signal calculated by ILC to enforce tracking of $\Phi^{*}(t)$ by Φ(t) (ILC also adjusts parameters in the feedback controller). The remaining parameters are described in the text, with full details given in [22]. Note that patient’s voluntary effort can be included as an external disturbance [36].

Next a 6 axis sensor was attached to the extreme link of the robotic support and ES was applied to each muscle in turn. The resulting force, h(t), recorded by the sensor was then related to the torque vector developed by the muscles, τ(t), via the Jacobian matrix (see Figure 3). A model of the dynamic relationship, τ(u(t), Φ(t),)$\dot{\Phi }$(t)), linking a vector containing the stimulation pulsewidth (in microseconds, μs) applied to each muscle, u(t) and the resulting torque generated, τ(t), was then identified using algorithms described in [27]. The remaining parameters in the dynamic model comprise the inertial matrix β(Φ(t)), Coriolis matrix, $C(\Phi (t),\dot{\Phi }(t))$ together with the non-conservative torque matrix $F(\Phi (t),\dot{\Phi }(t))$ which accounts for joint stiffness, spasticity, gravity and the unweighing action of the robot. These terms can all be identified by applying ES to the muscles while moving the arm using the sensor, and using an optimisation procedure on the resulting signals, h(t) and u(t) (see [22] for full details). Due to time constraints, generic model parameters for, B(·)C(·) and F(·) were used for each participant.

The resulting model shown in Figure 3 is significantly more complex than the previous planar case due to kinematic redundancy, additional degrees of freedom, multivariable inputs/outputs, and under/over-actuation. To tackle this control problem has necessitated significant extension in the ILC algorithms employed compared with the planar case [22,23]. First the vector of ideal joint movement is specified and denoted by $\Phi^{*}(t)$. On the k^th^ repetition of the task, the vector of joint angle errors is then denoted $e_{k}(t)=\Phi^{*}(t)-\Phi_{k}(t)$. In choosing the stimulation to supply on trial k + 1, the action of ILC is then to minimise a quadratic objective function of the form

(1) $$J\left(u_{k+1}(t)\right)=\sum {}_{t=0,t_{S},2t_{S}\cdots t_{d}}\left\{e_{k+1}{\left(t\right)}^{T}Re_{k+1}(t)+{\left(u_{k+1}(t)-u_{k}(t)\right)}^{T}Q\left(u_{k+1}(t)-u_{k}(t)\right)\right\}$$

where $t_{s}$ is the sampling time and $t_{d}$ is the duration of each trial. Through selection of weighting matrices Q and R, this objective function allows the designer to specify the relative importance of error reduction, or on the amount of ES applied to assist the patient’s movement. The optimal solution directly yields parameters appearing in the feedback controller, together with the feedforward signal, $v_{k+1}(t)$, applied on the subsequent trial. Full details of the ILC algorithms used, together with full details of the model can be found in [22,23,28], and [29].

### Unassisted tracking tasks

Participants completed four unassisted tracking tasks immediately following set up, and at the end of each session. These tasks involved tracking a slowly moving sphere along the far distal, far middle, off-centre middle, and centre distal trajectories at 10 seconds duration, (see Figure 2). Participants attempted each unassisted tracking trajectory once (i.e., each task consisted of one trial). Participants received no ES. For each task, there was a five-second countdown prior to the commencement of each trial (presented both visually and verbally).

To provide a measure of tracking performance which could be compared across different tasks, the norm of the tracking error for each joint was calculated (if $e_{i,k}(t)$ denotes the i^th^ element of the vector $e_{k}(t)$ at time ‘t’, then the norm of the tracking error for the i^^th^ joint is given by $\sqrt{\sum {}_{t=0,t_{s},2t_{s}\cdots td}e_{i,k}{\left(t\right)}^{2}}$). This norm is then divided by the norm of the reference trajectory for that joint, calculated in a similar manner. The result was subtracted from 1 so that a ‘performance’ of 1 corresponded to perfect tracking, and a negative value indicated movement away from the desired trajectory.

### Assisted tracking tasks

Assisted tracking tasks were selected according to clinical need. As such, some trajectories may have been used more than once or not at all. During each task, ES was applied to the muscles to assist the participant’s tracking. For three of the participants, the triceps and anterior deltoid were trained simultaneously (participants 1, 3, and 5). However, for two participants (2 and 4), an adverse response was observed when both muscles were stimulated (i.e., a flexor synergy was observed, probably related to spasticity). In these cases, ES was mainly applied to one muscle at time (e.g., stimulation of triceps and then anterior deltoid). Participants were instructed to move their arm so that their hand kept pace with the sphere. To indicate good performance, the sphere changed colour depending on error: green indicated tracking error of less than 5 cm and red indicated tracking error that was greater than 5 cm.

In each task, participants completed 6 trials tracking the same trajectory. A 15 second rest period between iterations was designed to reduce fatigue, and was extended if necessary. During this period a graphic was presented illustrating tracking performance for the trial just completed. The ILC calculated the optimal stimulation signals for application in the next iteration by minimising the objective function (1) using knowledge of the biomechanical model in combination with data from previous attempts. Participants started each movement from the same initial position, which was determined at the start of the first trial. Participants completed between 4–6 tasks in each session depending on fatigue.

**Figure 4:**
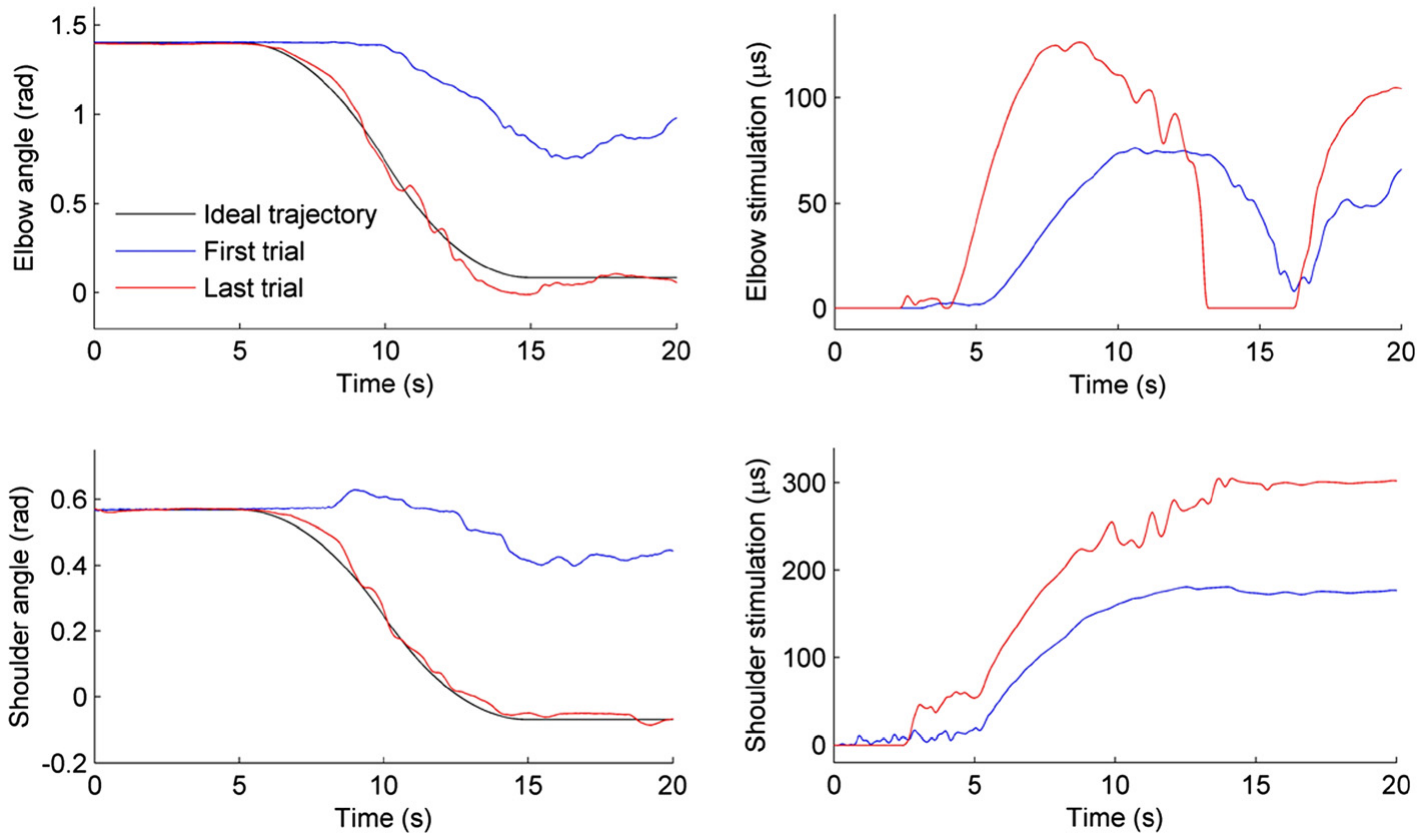
**Example of ILC correcting tracking: Elbow and shoulder tracking is shown on first and last trial (left-hand column), together with corresponding applied ES (right-hand column).** Five second padding is applied at beginning and end.

For each trial tracking performance was measured (as above) and the percentage of maximum ES applied was calculated by dividing the norm of the ES by the norm of the maximum stimulation that could be applied. Examples of these signals are shown in Figure 4, which also illustrates ILC correcting the applied ES to bring about accurate tracking.

## Statistical analysis

The data from the two pre-intervention assessment sessions were tested for differences using a *t*-test and then averaged for baseline performance. A one-tailed, paired *t*-test, with a significance level of *p* < .05, was used to compare baseline and post-intervention F-M and ARAT outcome measures. An improvement of 10% of the total number of points available for these measures was considered a clinically relevant improvement [34]. The maximum score for the F-M (motor component) was 66 and the maximum score for the ARAT was 57. In line with previous work [24,37], changes in assisted and unassisted performance were analysed by calculating best-fit linear regression slopes of performance against session number for each participant, and applying one-sample *t*-tests. Significance was associated with a value of *p* < .05. Clinical assessment data (means and standard deviations) and regression analyses (mean slopes and p-values) are given in Tables. Tracking performance is presented graphically.

## Results

### Participants

The five participants (three men and two women) were aged between 33 and 67 years (M = 52.6, SD = 15.27). Participants had suffered ischemic strokes, between 6 years 6 months and 11 months prior to recruitment to the study (M = 3 years 10 months, SD = 2 years); four had a hemiparesis of the left side and one of the right. All participants were right-side dominant prior to their stroke. All five participants complied with the study protocol (i.e., attended all sessions) and there was no withdrawal. During each intervention session participants spent 40–50 minutes practising reaching movements.

As described above, to reduce flexor synergy, stimulation was mainly applied to one muscle at a time for two participants (though note that both muscles were stimulated and trained). One of these participants reported experiencing temporary muscular aches in the hand and wrist, which was due to excessive gripping associated with the effort produced to move the impaired arm. Another participant reported experiencing minor discomfort around the shoulder which was associated with using the system and lasted about 1 week before disappearing. The other participants reported no adverse effects apart from minor fatigue following the intervention sessions.

### Clinical outcome measures

The clinical scores for the F-M and ARAT at baseline and after 18 intervention sessions are shown in Table 1. There were no significant differences between the two baseline assessment sessions for the F-M, *t*(4) = −2.08, *p* = .11, or ARAT, *t*(4) = −1.83, *p* = .14. A significant improvement was found from baseline to post-intervention for the F-M, *t*(4) = −4.54, *p* = .001, with all participants showing an improvement on the motor subtest of this assessment. This improvement was above the suggested 10% increase for clinical relevance in 3 of the 5 participants (see Table 1), although overall the 14% change was not statistically different from 10%, *t*(4) = 1.32, *p* = .26. No changes were found for the ARAT, *t*(4) = −.34, *p* = .37. Thus, the SAIL system reduced motor impairment of the upper arm in stroke participants but this did not transfer to functional improvements assessed by the ARAT.

**Table 1:** Assessment scores for the ARAT and F-M at baseline and post-intervention sessions

**P. Id**^**c**^	**ARAT (57 **^**a**^**)**		**F-M (Motor; 66**^**b**^**)**		
	**Baseline (pre-1, pre-2) average**^**d**^	**Post**	**Baseline (pre-1, pre-2) average **^**d**^	**Post**	**Change**
01	(0, 0) 0	1	(7, 12) 9.5	20	16%
02	(4, 10) 7	10	(19, 19) 19	33	21%
03	(9, 9) 9	10	(28, 34) 31	44	20%
04	(3, 5) 4	0	(15, 17) 16	21	8%
05	(11, 13) 12	13	(42, 42) 42	46	6%
Mean(SD)	6.4 (4.62)	6.8 (5.89)	23.5 (12.95)	32.8 (12.28)	14%

Note: ^a^ maximum score for hemiplegic side; ^b^ maximum score for motor component of the assessment; ^c^ P.Id. = participant identity number; ^d^Baseline = pre-1 and pre-2, refer to the scores from the two pre-intervention assessments. The number outside the parentheses = average score collapsed over the two pre-intervention assessments.

### Unassisted tracking performance

**Figure 5:**
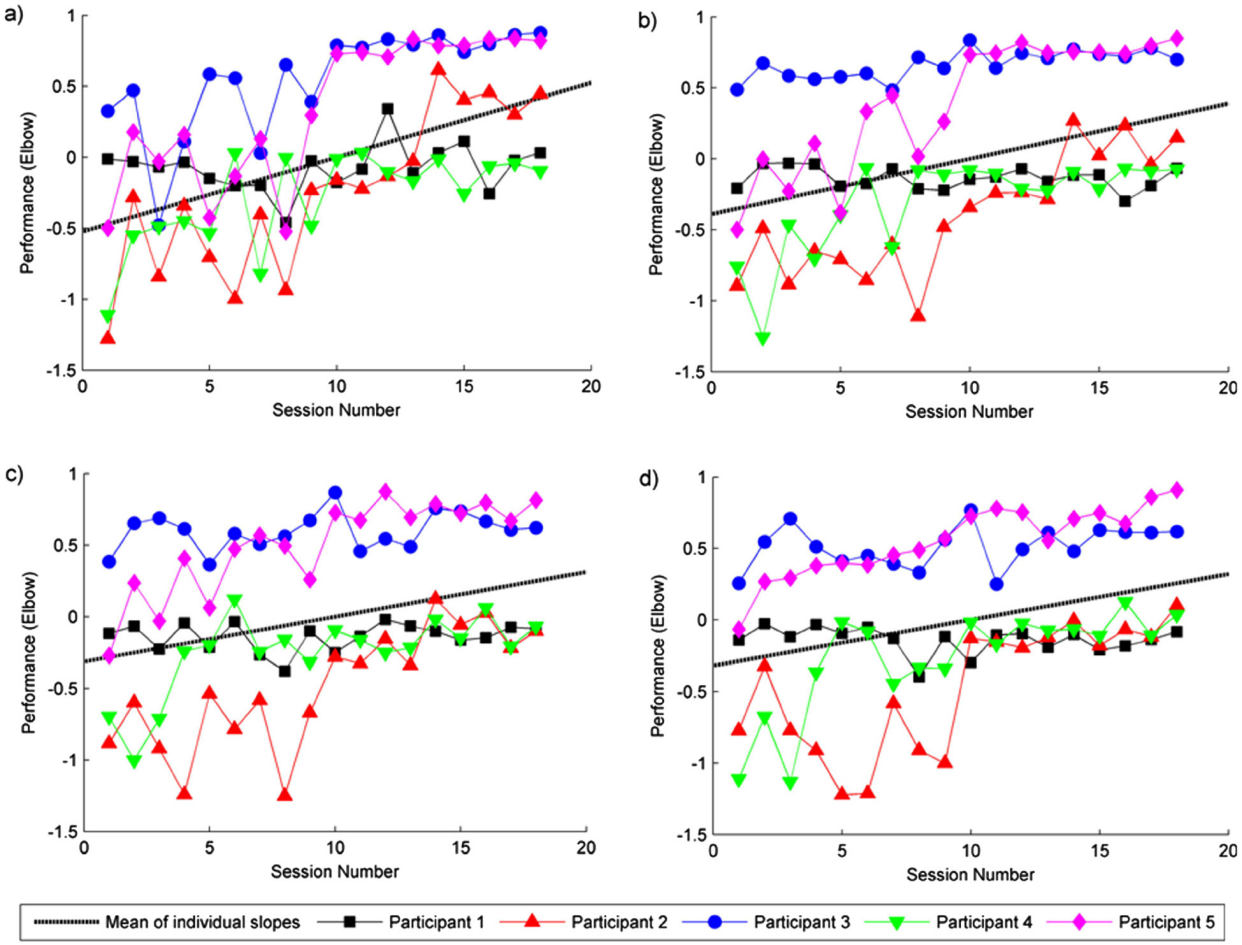
**Unassisted tracking performance for the elbow as a function of session, for each participant and unassisted task.** Panel **a)** shows tracking performance for the centre-distal task; Panel **b)** shows tracking performance for the off-centre-middle task; Panel **c)** shows tracking performance for the far middle task; Panel d) shows tracking performance for the far distal task. 1= perfect tracking performance. Best-fitting regression slopes were calculated for each combination of participant, muscle and task, with mean slopes (across participants) shown

Figure 5 illustrates unassisted tracking performance for the elbow as a function of session, for each participant and task. Similar patterns of performance were found for the shoulder. Best-fitting regression lines were calculated for each combination of participant, task, and muscle (giving 40 slopes in total), and one-tailed t-tests found that the slopes (collapsed across all participants) were reliably positive for each of the four unassisted tasks for both the shoulder and the elbow (see Table 2). That is, the slopes were significantly different from 0, showing that tracking accuracy (i.e., error between arm position and target) improved over the course of the intervention for both shoulder and elbow movements. Note that the mean slopes in Table 2 correspond to performance increases of between 49% and 93% over the course of the intervention, thereby confirming significant improvement.

**Table 2:** Mean slope (and p-value) of the best fit regression lines (collapsed across participants) for performance measures in the unassisted and assisted SAIL tracking tasks

	**Elbow**		**Shoulder**	
	**Slope**	**p-value**	**Slope**	**p-value**
	Unassisted Performance measures			
Centre distal trajectory	0.053	.01	0.055	.05
Off-centre middle trajectory	0.039	.03	0.034	.02
Far middle trajectory	0.031	.03	0.029	.01
Far distal trajectory	0.032	.03	0.032	.007
	Assisted Performance measures			
Assisted tracking performance	.010	.03	.012	.01
Max. % of ES applied	−1.306	.02	−1.370	.02
Performance/ES	.0008	.03	.0013	.03

Note: small slope values are due to differences in axes units. See Figures 5 and 6 for a graphical representation of slopes.

### Assisted tracking performance

**Figure 6:**
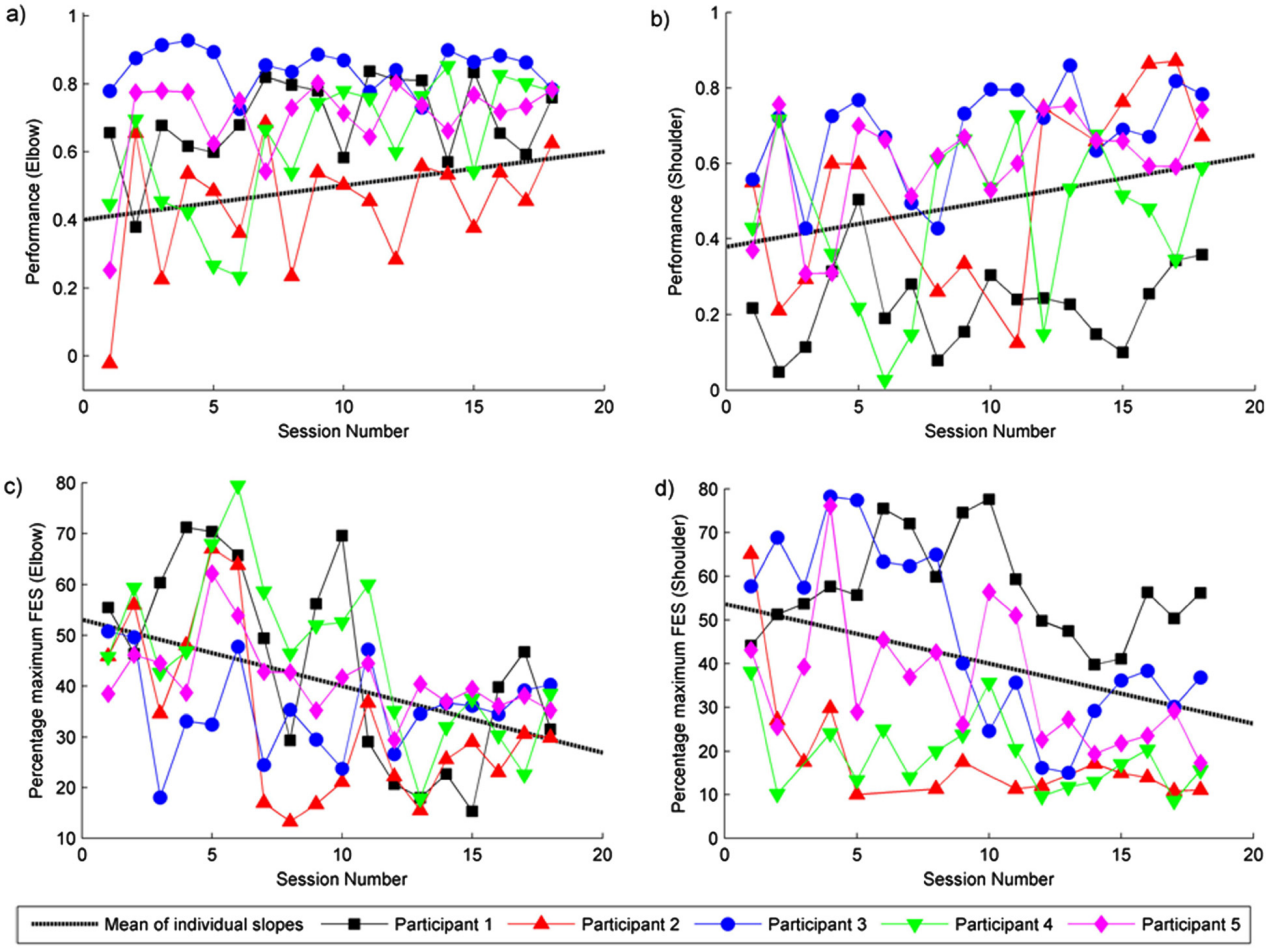
**Panel a) and b) show tracking performance in the assisted tasks over sessions for the elbow and the shoulder respectively; Panel c) and d) show the percentage maximum stimulation applied to the triceps and anterior deltoid respectively, over sessions.** Best-fitting regression slopes were calculated for each combination of participant and muscle, with mean slopes (across participants) shown.

Tracking performance measures and the percentage of maximum stimulation applied were calculated using the final trial in each task and were averaged across tasks in each session. As shown in Figure 6, for both the shoulder and the elbow, participants tracking performance became more accurate over the 18 sessions, and the percentage maximum stimulation decreased. Best-fitting regression lines were calculated for each participant and muscle, and one-tailed, one-sample *t*-tests found that the slopes collapsed across all participants were statistically significant for each muscle (see Table 2). This suggests that the amount of movement produced by the ES, for both the triceps and anterior deltoid, increased over the intervention. To further qualify this, we divided tracking performance from the final trial in each task by the corresponding percentage maximum stimulation, and averaged across tasks in each session. The slopes of the best-fitting regression lines were found to be significantly positive (see Table 2), confirming that over the intervention a greater amount of performance is elicited per unit of ES applied. Note that the small mean slope values in Table 2 are due to the difference in scale of the units used to measure ES and performance.

## Discussion

The main aims of the study were to investigate the feasibility and effectiveness of SAIL, a novel 3D stroke rehabilitation platform for the upper limb that combines ES mediated by ILC, robot and VR technology. This system uses the most advanced model-based ES controllers that have been employed clinically in upper arm stroke rehabilitation, and comprises a substantial development upon previous use of ILC in this area. The effectiveness of ES is suggested to be most beneficial when combined with a person’s own voluntary intention to move [12]. The ILC component of SAIL was employed to optimise the potential benefit of this. Three key findings confirmed SAIL feasibility and effectiveness from baseline to post-intervention: a clinically significant improvement in the F-M; an improvement in unassisted tracking performance; and a reduction in the amount of ES required for accurate assisted tracking.

Tracking performance in the assisted tasks was more accurate than tracking in the unassisted tasks. In addition, we found a reduction in the amount of ES applied to the muscles, and an increase in the accuracy of assisted tracking. This demonstrates that ILC mediated ES can assist participants in making precise reaching movements, and confirms the feasibility of SAIL with chronic stroke participants. Further tests are now required to determine the relative contribution of muscle strength and voluntary control to improved tracking performance to explain the reduction in ES.

The results showed improvements in unassisted tracking performance over the course of the intervention and improvements in F-M scores. These performance measures indicated that training the triceps and anterior deltoid (though not always at the same time for two participants) using SAIL improved movement of the upper limb in five stroke participants. However, the observed motor improvement did not transfer to functional improvements, as measured by the ARAT. This is consistent with previous work, with a number of systematic reviews reporting that robotic therapy reduces motor impairment but does not improve functional impairment [8,13,14,16]. The ability to use the hand is an integral component of the ARAT and other functional outcome measures. As the SAIL system only trained the triceps and anterior deltoid this may explain why no change was found on this outcome measure. This finding implies that to observe changes in functional outcome measures, future work should extend the application of this intervention to the hand and wrist. As the movement complexity increases, there is more emphasis on model-based approaches to provide optimal performance which maximises effectiveness of therapy, and work by the authors is underway to address these issues.

Current findings were also in line with those of the previous study, in which ILC mediated ES was used to assist stroke participants in planar reaching movements [24]. Specifically, Hughes et al. [24] found an increase in tracking performance, a reduction in applied ES, and a marginal improvement in F-M scores (although improvements were less than 10%). The observed improvements in the F-M scores were greater in the current compared to the previous study (mean difference of 9.3 vs. 2.5; see [24]). Furthermore, the observed F-M improvement was greater than 10% (although not significantly so), indicating a trend towards clinical relevance [34]. One possible reason for the difference in results is that the current SAIL intervention trained two muscles in 3D space, whereas the previous intervention trained only triceps in 2D space. Alternatively, participants in the current study had higher initial F-M scores than those reported by Hughes et al. [24] and this may have contributed to the differences found.

As with any study, there were limitations. For example, due to the 3D element to the task and the complexity of shoulder movements, there was more variability in participants’ movement. This made it difficult to get consistent responses in each of the intervention sessions, especially for the shoulder. In addition, the sample size was small and there was no control condition. Therefore, caution must be taken when generalising the results, as possible confounding effects such as age or spasticity were not considered and it is difficult to determine whether the effects found were due to the unique ILC component, or were simply due to practice. In addition, although significantly different from 0, the mean values for Performance/ES shown in Table 2 are small. This is mainly due to the difference in axes units used, but the small sample size means that caution should be taken regarding clinical significance. It is important to note, however, that the focus of this study was to demonstrate feasibility of this technique, and our main findings do confirm the concept of minimising support from ES using ILC algorithms. To verify and extend these results, future work should test the intervention in a larger scale project, including more participants and a control condition in which ES is used without ILC.

## Conclusions

In summary, this feasibility study has demonstrated the potential impact for the technology used in SAIL. The technology provides rehabilitation that is tailored to an individual’s need and can be easily transferred between different rehabilitation platforms, which could be used to increase the intensity of practice and stimulate muscles in the whole arm. In this way, with further development to a portable device, SAIL may be viable for use in home settings. The technology employed by SAIL was designed to help stroke patients train their upper limb muscles during reaching tasks, to improve motor control. The results from this study demonstrate the feasibility of using ILC to mediate ES to assist precise upper limb movements. Three key findings confirmed this: There were significant improvements in F-M scores and tracking performance, and a reduction in the amount of ES required for accurate assisted tracking. In conclusion, SAIL can assist upper limb movement training in chronic stroke participants, minimizing ES support whilst maintaining accurate movements. The positive results indicate that the application of SAIL technology may be clinically relevant for chronic stroke rehabilitation and are promising with respect to reducing upper limb impairment.

## Abbreviations

ES, Electrical Stimulation; ILC, Iterative Learning Control; SAIL, Stimulation Assistance through Iterative Learning; VR, Virtual Reality; F-M, Fugl Meyer; ARAT, Action Research Arm Test.

## Competing interests

JH is a board member of Hocoma. The authors declare no other competing interests.

## Authors’ Contributions

KM participated in the design and coordination of the study, participated in acquisition and analysis of data and drafted the manuscript; AH participated in the design of the study, led the clinical aspects of the study, and made substantial contributions to the revision of the draft; CF conceived the concept of the study and led the engineering design and development, performed statistical analysis, and made substantial contributions to the revision of the draft; DT and ZC participated in the design and development of the VR rehabilitation platform and data acquisition; ER and JB provided consultation on the engineering and clinical aspects of the study respectively. All authors have read and approved the final manuscript.
